# The epigenetic effects of butyrate: potential therapeutic implications for clinical practice

**DOI:** 10.1186/1868-7083-4-4

**Published:** 2012-02-27

**Authors:** Roberto Berni Canani, Margherita Di Costanzo, Ludovica Leone

**Affiliations:** 1 Department of Pediatrics, University of Naples 'Federico II', Via S Pansini 5, Naples 80131, Italy; 2 European Laboratory for the Investigation of Food Induced Diseases, University of Naples 'Federico II', Via S Pansini 5, Naples 80131, Italy

**Keywords:** epigenome, histone deacetylase inhibitor, short chain fatty acids

## Abstract

Butyrate is a short chain fatty acid derived from the microbial fermentation of dietary fibers in the colon. In the last decade, multiple beneficial effects of butyrate at intestinal and extraintestinal level have been demonstrated. The mechanisms of action of butyrate are different and many of these involve an epigenetic regulation of gene expression through the inhibition of histone deacetylase. There is a growing interest in butyrate because its impact on epigenetic mechanisms will lead to more specific and efficacious therapeutic strategies for the prevention and treatment of different diseases ranging from genetic/metabolic conditions to neurological degenerative disorders. This review is focused on recent data regarding the epigenetic effects of butyrate with potential clinical implications in human medicine.

## Introduction

The intestinal microbiota plays a critical role in the establishment and maintenance of body health. Commensal bacteria are involved in the fermentation of dietary fibers in the colon leading to production of short chain fatty acids (SCFAs), 2-carbon to 5-carbon weak acids including acetate (C2), propionate (C3), butyrate (C4) and valerate (C5). Among the SCFAs, butyrate has received particular attention for its multiple beneficial effects from the intestinal tract to the peripheral tissues [1]. The most important butyrate producers appear to be *Faecalibacterium prausnitzii*, which belongs to the *Clostridium leptum *(or clostridial cluster IV) cluster, and *Eubacterium rectale/Roseburia *spp., which belong to the *Clostridium coccoides *(or clostridial cluster XIVa) cluster of firmicute bacteria [2]. The mechanisms of action of butyrate are multiple, but many of these are related to its regulatory effects on gene expression. Butyrate is part of a well known class of epigenetic substances known as histone deacetylase inhibitors (HDACi). Epigenetics focuses on the mechanisms that mold chromatin structures and regulate gene expression with stability, thus defining cell identity and function and adapting cells to environment, without changing the nucleotide sequence. There are three distinct, but closely interacting, epigenetic mechanisms (histone acetylation, DNA methylation, and non-coding microRNAs) that are responsible for modifying the expression of critical genes associated with physiologic and pathologic processes [3]. Histone tail acetylation is believed to enhance the accessibility of a gene to the transcription machinery, whereas deacetylated tails are highly charged and believed to be tightly associated with the DNA backbone, thus limiting accessibility of genes to transcription factors [4]. Modulation of histone acetylation and deacetylation through environmental factors, including dietary compounds, may prevent diseases and maintain health. In a broader context, there is a growing interest in dietary HDACi, in particular butyrate because its impact on epigenetic mechanisms will lead to more specific and efficacious therapeutic strategies in the prevention and treatment of different diseases. This review is focused on recent data regarding the epigenetic effects of butyrate with potential clinical implications in human medicine.

### Cancer therapy and chemoprevention

Recently, in the area of cancer biology increasing attention has been given to the role of epigenetic alterations in the etiology of cancer. A particularly active field of research involves HDACi, not only for cancer therapy but also for cancer chemoprevention. In contrast to genetic defects, epigenetic aberrations can be reversible: a trait that represents a key aspect of their potential as therapeutic targets. HDACi cause changes in the acetylation status of chromatin and other non-histone proteins, resulting in changes in gene expression, induction of apoptosis, cell cycle arrest, and inhibition of angiogenesis and metastasis [5,6]. Several HDACi are involved in various stages of development, including clinical trials as monotherapies and in combination with other anticancer drugs and radiation treatments [7,8]. However, the molecular mechanisms underlying the response to HDACi in cancer patients are not fully understood. Butyrate and phenylbutyrate are on the market for non-oncologic uses for years and have been shown to have HDACi activity. These SCFAs have HDACi activity at millimolar concentrations. The ability of butyrate to de-repress epigenetically silenced genes in cancer cells, such as cell cycle inhibitor p21 and the proapoptotic protein Bcl-2 homologous antagonist/killer (BAK), and to activate these genes in normal cells, has important implications for cancer prevention and therapy [9]. HDACi can also induce autophagic cell death. HeLa cells with apoptotic protease activating factor 1 (Apaf-1) knockout or B cell lymphoma-extra large (Bcl-XL) overexpression were induced to autophagic cell death with autophagic vacuoles in the cytoplasm, when cultured with butyrate [10,11]. Furthermore, HDACi have been described to have antiangiogenic and antimetastatic effects. Butyrate was found to repress angiogenesis *in vitro *and *in vivo*, and reduce expression of proangiogenesis factors, including hypoxia inducible factors (HIF-1a) and vascular endothelial growth factor (VEGF) [12-14]. Several studies have hypothesized that an increased colonic concentration of butyrate can be an important mediator in the observed protective effect of fermentable dietary fibers against colorectal cancer [15,16]. The protective role of dietary fibers and their breakdown product butyrate against colorectal cancer could be determined by a modulation of canonical Wnt signaling, a pathway constitutively activated in the majority of colorectal cancers [17]. Butyrate is recognized for its potential to act on secondary chemoprevention by slowing growth and activating apoptosis in colon cancer cells, but it can also act on primary chemoprevention [18]. The mechanism proposed is the transcriptional upregulation of detoxifying enzymes, such as glutathione-S-transferase (GST). The modulation of the GST gene may protect cells from genotoxic carcinogens, such as H_2_O_2 _and 4-hydroxynonenal (HNE) [19,16]. A new compound named pivanex (pivaloyloxymethil butyrate) has demonstrated an antineoplastic activity in the treatment of non-small-cell lung cancer. Its effect is based on a rapid hydrolysis and release of butyrate, permitting efficient delivery to subcellular targets. Pivanex has been shown to be active and safe in pretreated non-small-cell lung cancer patients and these interesting results led this drug to be administered in combination with docetaxel [20].

### Anti-inflammatory effects

The principle mechanisms through which butyrate exerts its anti-inflammatory effects are the suppression of nuclear factor κB (NFkB) activation, the inhibition of interferon γ production and the upregulation of peroxisome proliferator-activated receptor γ (PPARγ), which may result from the inhibition of HDAC [21]. These properties of butyrate led us to evaluate its possible therapeutic use in inflammatory bowel disease (IBD). Intervention studies in patients with ulcerative colitis (UC) suggested that the luminal administration of butyrate or stimulation of luminal butyrate production by the ingestion of dietary fibers results in an amelioration of the inflammation and symptoms [22,23]. Hallert *et al*. instructed 22 patients with quiescent UC to add 20 g of dietary fibers to their daily diet. A total of 4 weeks of this treatment resulted in a significant increase of fecal butyrate concentration and in a significant improvement of abdominal symptoms [22]. Vernia *et al*. in a double-blind, placebo-controlled multicenter trial, treated 51 patients with active distal UC with rectal enemas containing either 5-aminosalicylic acid (5-ASA) or 5-ASA plus sodium butyrate (80 mM, twice a day). The combined treatment with topical 5-ASA plus sodium butyrate resulted in a significant improvement of the disease activity score compared to that observed in patients treated with 5-ASA alone [23].

### Effects on obesity, insulin resistance and cardiovascular diseases

The anti-inflammatory effect of butyrate, mediated by inhibition of HDAC, may also be able to prevent the infiltration of immune cells from the bloodstream in the adipose tissue [24]. In this regard, it could be interesting to evaluate butyrate in the prevention/treatment of chronic low grade inflammation, frequently seen in obesity and associated with an increased risk of insulin resistance, type 2 diabetes, and cardiovascular diseases. In a preclinical study, Gao *et al*. showed that dietary supplementation of butyrate can prevent and treat diet-induced obesity and insulin resistance in mouse models. Butyrate acts through a stimulation of peroxisome proliferator-activated receptor-γ coactivator 1α (PGC-1α) activity, which results from the inhibition of HDAC and the activation of AMP-activated protein kinase (AMPK) [25]. In hypercholesterolemia, recent evidence shows that the global effect of butyrate is to downregulate the expression of nine key genes involved in intestinal cholesterol biosynthesis, thus possibly inhibiting this pathway [26]. These data suggest that butyrate may have potential application in the prevention and treatment of the main components of the so-called metabolic syndrome in humans. Moreover, butyrate showed to have a more direct protective role in cardiovascular diseases. Recently, Mathew *et al*. demonstrated that butyrate exhibits atheroprotective/antiatherogenic potential by altering G1-specific cell cycle proteins through its chromatin remodeling activity to arrest vascular smooth muscle cells proliferation, a critical cellular component of the blood vessel, that play a major role in the development of atherosclerosis. The results of this study suggest a potential role for butyrate both in cardiovascular disease prevention and in therapeutic intervention of arterial restenosis and in-stent restenosis as a pharmacological agent, respectively [27].

### Effects on the immune system

Another emerging area of research involves the epigenetic regulation of the immune system exerted by butyrate. Epigenetic pathways act at many levels to regulate immune functions: in immune system development, in response to infection, in immune surveillance to tumors and in autoimmunity [28]. In particular, butyrate seems to exert broad anti-inflammatory activities by affecting immune cell migration, adhesion, cytokine expression as well as affecting cellular processes such as proliferation, activation and apoptosis [24]. The effects of butyrate are also exerted on the innate immune system. The antimicrobial protein cathelicidin plays an important role in the defense mechanisms against bacterial infection. Several studies show that sodium butyrate induces cathelicidin gene expression in human colonic, gastric and hepatic cells [29-31]. Kida *et al*. investigated the mechanisms involved in sodium butyrate-induced cathelicidin gene expression in a human lung epithelial cell line, EBC-1. This study indicated that sodium butyrate could induce both the cathelicidin mRNA and protein expression. Furthermore, they demonstrated that activator protein 1 (AP-1) and histone acetylation of cathelicidin promoter participate in the regulation of inducible cathelicidin gene expression [32]. Thus, the use of HDACi to enhance the expression of cathelicidins may become a novel approach of strengthening innate immunity.

### Effects on inherited disorders

Butyrate can also be considered an inducer of fetal hemoglobin (HbF) synthesis, an interesting strategy for the treatment of β-hemoglobinopathies, such as sickle cell disease and β-thalassemia. The induction of HbF synthesis involves the epigenetic regulation of γ-globin gene expression, which is mediated by HDAC inhibition. Clinical trials in patients with sickle cell disease and β-thalassemia confirmed the ability of butyrate to increase HbF production [33,34].

Butyrate and its derivatives have been investigated as a potential approach for the treatment of cystic fibrosis [35]. Class II mutations of cystic fibrosis transmembrane conductance regulator (CFTR) can be restored to the protein trafficking pathway by manipulation of chaperone protein/CFTR interactions with chemical chaperones or drugs that affect gene regulation such as butyrate. Investigations have demonstrated *in vitro *activity in the form of increased production of mature CFTR and chloride transport at the cell surface. The mechanism is still not fully understood but is likely to involve upregulation at the transcriptional level and modulation of protein folding step. Butyrate is likely to show activity for other inherited disorders with alternative enzymatic pathways that could be upregulated, such as X-linked adrenoleukodystrophy (X-ALD), a disorder of peroxisomes due to mutations in ABCD1 gene and characterized by altered metabolism and accumulation of very long chain fatty acids. Histone deacetylase inhibitors reduce the oxidative damage in X-ALD cells, moreover they could induce the expression of the closest homolog of ABCD1, the redundant gene ABCD2, which should be able to compensate for the lack of functional ABCD1 in X-ALD patients [36]. Congenital chloride diarrhea (CLD) is another inherited disorder in which butyrate therapy has been shown to be beneficial [37,38]. CLD is a rare genetic disease caused by mutations in the gene encoding the solute-linked carrier family 26-member A3 (SLC26A3) protein, which acts as a plasma membrane anion exchanger for Cl^- ^and HCO_3_^-^. The mechanism underlying this therapeutic effect could be related to stimulation of the Cl^-^/butyrate exchanger activity, to reduction of mistrafficking or misfolding of the SLC26A3 protein, or alternatively butyrate may induce the expression of SLC26A3 gene, which contains a 290-bp region between residues -398 and -688 that is crucial for high-level transcriptional activation induced by butyrate. This may explain the variable response of patients affected by CLD to butyrate; in fact, depending on the patient's genotype, mutations in the above-mentioned regulatory regions of the SLC26A3 gene could affect gene transcription rate.

### Neuroprotective effects

A new field of application of butyrate is in ischemic stroke: butyrate seems to have long-term beneficial effects after ischemic injury. Cerebral ischemia enhances neurogenesis in neurogenic and non-neurogenic regions of the ischemic brain of adult animal models. A preclinical study demonstrated that post-insult treatment with sodium butyrate stimulated cell proliferation, migration and differentiation through the upregulation of the brain-derived neurotrophic factor (BDNF) in the ischemic brain of rats subjected to permanent cerebral ischemia [39]. Butyrate also exerts significant neuroprotective effects in a transgenic mouse model of Huntington's disease (HD), and therefore represents a very promising therapeutic approach for HD. There is evidence that mutant Huntington protein interacts with transcription factors leading to reduced histone acetylation. Administration of the HDACi phenylbutyrate after onset of symptoms in a transgenic mouse model of HD significantly extends survival and attenuates neuronal atrophy [40]. Other interesting effects of butyrate are those that were observed on memory. Blocking HDAC activity with non-specific HDACi, such as sodium butyrate, enhances synaptic plasticity and memory, suggesting that HDACs may actually serve to return chromatin to a repressive state and silence transcription required for long-term memory formation [41]. Moreover, HDAC inhibition can transform a learning event that does not normally lead to long-term memory for object recognition into a long-lasting form of memory [42]. Together, these results suggest HDACs may serve as critical memory suppressor genes and show that HDACi, such as butyrate, may generate more persistent forms of long-term memory, which has great therapeutic and translational value.

### Effects on stem cells

Butyrate greatly enhances the efficiency of induced pluripotent stem cell derivation from human adult or fetal fibroblasts by promoting epigenetic remodeling and the expression of pluripotency-associated genes [43]. Thus, butyrate as a cell permeable small molecule provides a simple tool to further investigate molecular mechanisms of cellular reprogramming. Moreover, butyrate stimulation provides an efficient method for reprogramming various human adult somatic cells, including cells from patients that are more refractory to reprogramming. Different evidence suggests that cancer stem-like cells exist in several malignant tumors, such as leukemia, breast cancer, and brain tumors, and that these stem cells express surface markers similar to those expressed by normal stem cells in each tissue [44-46]. In a recent study, Kato *et al*. isolated side-population cells and non-side-population cells derived from a rat endometrial cell line expressing human [^12^Val] KRAS (RK12V cells) and determined the side-population phenotype. RK12V-SP cells showed self-renewal capacity, the potential to develop into stromal cells, reduced expression levels of differentiation markers, long-term proliferating capacity in cultures, and enhanced tumorigenicity, indicating that RK12V-SP cells have cancer stem-like cell features. RK12V-SP cells also display higher resistance to conventional chemotherapeutic drugs. In contrast, treatment with sodium butyrate reduced self-renewal capacity and completely suppressed colony formation of RK12V-SP cells in a soft agar, demonstrating an inhibitory effect of sodium butyrate on proliferation of endometrial cancer stem-like cells [47].

## Conclusions

**Figure 1:**
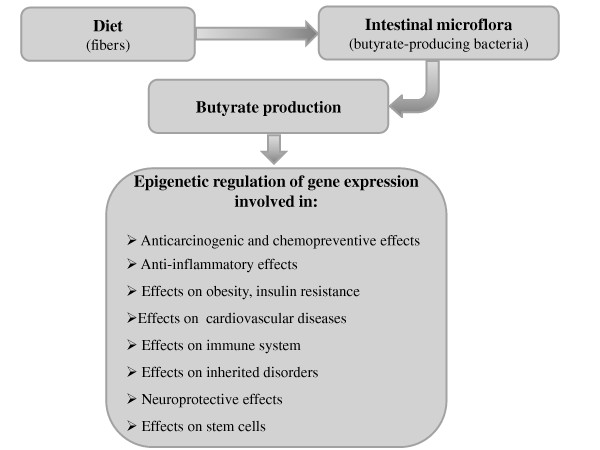
**Diet influences intestinal microflora composition, which has an important role in the fermentation of dietary fibers leading to the production of short chain fatty acids (SCFAs), such as butyrate**. Butyrate exerts multiple beneficial effects at intestinal and extraintestinal level, related at least in part to the epigenetic regulation of gene expression.

The gastrointestinal tract contributes to body health in many ways. Of particular interest in this context is the emerging concept of 'gut health' and the mechanisms that can explain it. 'Gut health' is a term increasingly used in the medical literature. It covers multiple positive aspects of the gastrointestinal tract, such as the absence of gastrointestinal illness, normal and stable intestinal microbiota, and effective immune status, that are also able to determine a state of well-being [48]. There is now ample evidence that two functional entities are crucial to achieve and maintain 'gut health' [49,50]. These entities are the intestinal microbiota and the intestinal barrier. Any impairment of the intestinal microbiota, for example, deriving from an unbalanced diet such as a carbohydrate-rich diet, could affect gut functionality and consequently 'gut health' [51]. Thus, 'gut health' can offer a new approach for preventive medicine if we learn more about how to achieve and maintain it. Lifestyle characteristics, such as a balanced diet, moderate but regular exercise and avoidance of chronic stress, but also defined products such as select prebiotics and probiotics, can support gut health [48]. In this context, the important role of intestinal microflora in the production of SCFAs from the earliest stages of life has lead to the idea that probiotics represent a novel therapeutic or preventive strategy for chronic diseases, restoring SCFA levels by modulating the microbial environment (Figure 1) [52]. More recently, Licciardi *et al*. hypothesized that the epigenetic mechanisms elicited by probiotics through the production of SCFAs, especially butyrate, are the key to understand how they mediate their numerous health promoting effects from the gut to the peripheral tissues [53]. Butyrate has a pivotal role in the context of 'gut/body health'. Its production is dependent on diet and intestinal microflora composition, but it is also able to modulate intestinal microflora through regulation of lumen pH and to exert many beneficial extraintestinal effects through epigenetic mechanisms [52]. In a broader context, butyrate may be useful in the prevention and treatment of different chronic disorders, including cancer, metabolic syndrome, cardiovascular diseases, inherited disorders, immune-mediated chronic disorders, and neurodegeneration. Moreover, butyrate might help with developing improved strategies for regenerative medicine by promoting epigenetic remodeling and the expression of pluripotency-associated genes. In conclusion, numerous preclinical data have been obtained in the last decade, and these findings provide the basis for future clinical studies aiming to elucidate the potential preventive and therapeutic role(s) of butyrate in human medicine.

## Competing interests

The authors declare that they have no competing interests.

## Authors' contributions

BCR and DCM were responsible for the conceptualization and implementation of the manuscript. BCR, DCM and LL were responsible for writing the manuscript. All authors read and approved the final manuscript.
